# Comprehensive Approach to Atomistic Simulations of Discharging Batteries

**DOI:** 10.1021/acs.jctc.6c00702

**Published:** 2026-08-06

**Authors:** Pramudit Tripathi, Scott T. Milner

**Affiliations:** † Department of Chemical Engineering, The Pennsylvania State University, University Park, Pennsylvania 16802, United States; ‡ Department of Materials Science and Engineering, The Pennsylvania State University, University Park, Pennsylvania 16802, United States

## Abstract

Predicting the performance of batteries using analytical and computational models plays an important role in the design of battery packs and management systems. Currently, these models rely on extensive experimental parametrization; but fundamentally, these parameters arise from atomistic interactions between components of the electrolyte and the resulting correlated motion of the ions. In this work, we demonstrate a comprehensive approach to atomistic simulations of discharging batteries, evaluating electrochemical potential gradients in the electrolyte and using Onsager mobility coefficients to relate the resulting forces to the flux of lithium ions between the electrodes. This work unifies four different sets of simulations: (1) mobility, which observes the molecular flux of species in response to constant forces; (2) thermodynamic susceptibility, which observes the response of species concentration to external potentials; (3) bulk modulus, which observes the density response to pressure; and (4) battery discharge, which generates a steady flux of cations and observes the resulting concentration and potential gradients. The resulting model self-consistently describes ion-transport battery electrolytes.

## Graphical abstract

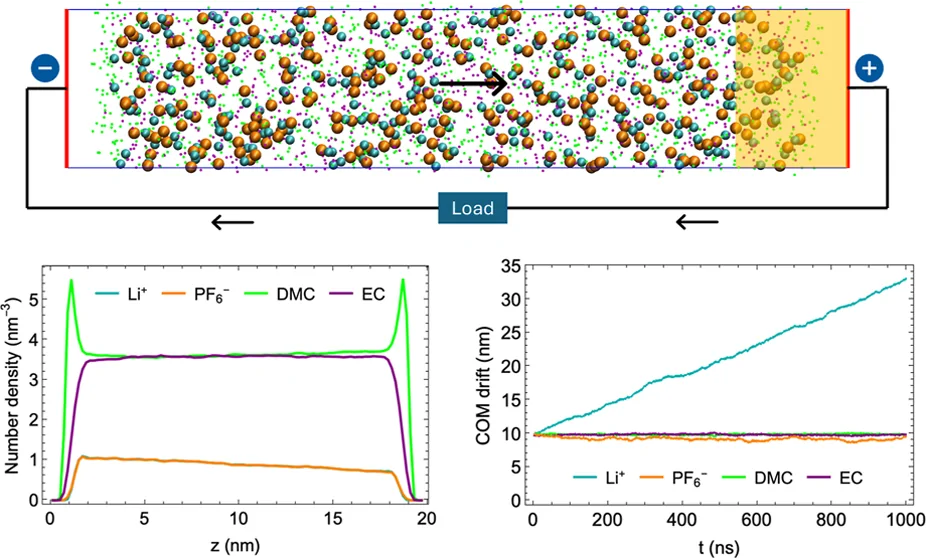

## 1 Introduction

The performance of lithium-ion batteries depends on the facile transport of lithium ions through the electrolyte. When a battery discharges, lithium ions migrate from the negative to the positive electrode, while counterions and solvent molecules are physically confined between the electrodes. Each species experiences forces arising from an electric field, concentration gradients, and mechanical stresses. The mesoscopic behavior that we seek to predict includes ionic conductivity, discharge rates, concentration profiles, and electric potential distributions across the cell. Note that these properties depend on correlated motion of all electrolyte species and not simply on the drift of ionic species. The resulting ion transport determines the ionic conductivity, transference number, internal voltage drop, and discharge rate.

The continuum approach to battery modeling, exemplified by the Doyle–Fuller–Newman (DFN) model, describes battery discharge using conservation laws for charge and mass applied at the cell scale.^1,2^ This model employs concentrated solution theory^3^ to couple cation and anion transport and requires more than 30 empirical parameters to fully describe a battery cell. Over the years, improvements have been made to continuum models, which focus on improved accuracy or reduced computational cost.^4–6^ This framework has been used to predict discharge curves and optimize battery designs.^7^

Evidently, continuum models require extensive parametrization from experiments, many of which require invasive measurements of batteries in operation, which limits their applicability.^7^ Furthermore, the continuum approach provides no insight into the molecular origins of the many parameters or the microscopic mechanisms that generate them. This gap motivates a complementary, molecular-scale approach.

Molecular dynamics (MD) simulations have emerged as a powerful tool for predicting intrinsic transport properties of electrolytes.^7,8^ Classical MD simulations can quantitatively reproduce the ionic conductivity and diffusivity of battery electrolytes.^9–11^ In particular, Onsager transport coefficients, which describe the correlated motion of electrolyte species, can be efficiently extracted from nonequilibrium MD simulations, in which constant forces are applied to species, and the resulting drift velocities are observed.^10,12^

MD simulations can predict transport properties of typical lithium-ion electrolytes in quantitative agreement with experiments, while revealing atomistic details of ion solvation and clustering.^8,10,11,13^ In particular, for widely used liquid electrolytes of lithium salts in organic carbonates, MD simulations accurately predict ionic conductivity, diffusivity, and preferential solvation.^10,14,15^ Further, MD simulations of electrode–electrolyte interfaces provide valuable insights into effects of electrified surfaces on ion solvation and dynamics.^16^

Hanke et al. parametrized the Doyle–Fuller–Newman model using ionic diffusivities extracted from MD simulations to predict battery discharge curves. They used the Nernst–Einstein equation to predict ionic conductivity from diffusivity, thereby inherently ignoring the correlated motion of ions in their approach. They also treated the discharge process at the continuum scale, leaving the atomistic mechanisms of discharge unexplored.

Atomistic simulations have not previously been developed to represent steady-state discharge in which a sustained flux of lithium ions develops across the electrolyte in response to forces arising from charge accumulation at the electrodes.

In this work, we develop a novel approach to simulate steady-state battery discharge at atomistic detail and compare its behavior to predictions from microscopic parameters likewise obtained from simulations. Our approach includes three sets of calibrating simulations: (1) measurement of the Onsager mobility matrix, by observing species drift in response to constant forces; (2) measurement of the thermodynamic susceptibility matrix, by observing species concentration response to external potentials; and (3) measurement of the bulk modulus, by observing the density response to pressure. Finally, we simulate battery discharge, in which electrodes absorb and release lithium ions, maintaining a steady flux from the negative to positive electrode.

From the concentration and potential gradients observed in the discharge simulations, we can use our thermodynamic susceptibilities to predict the force on each species. Then, from our Onsager mobilities, we can predict the resulting fluxes for each species, which we can compare to the fluxes observed in the discharge simulations, to demonstrate consistency with our thermodynamic and transport measurements.

We demonstrate this approach on 1.5 M LiPF_6_ in equimolar dimethyl carbonate (DMC) and ethylene carbonate (EC), which is a typical organic liquid electrolyte widely used in commercial batteries. This electrolyte is well characterized experimentally and has been extensively studied by MD simulations, making it an ideal test system. However, we emphasize that the methods developed here are applicable to any electrolyte.

This work describes the computational methods used to characterize the electrolyte—the three calibrating simulations and the battery discharge simulation in the Methods and Results section, followed by the analysis of forces acting on each electrolyte component and their origins in the Discussion section. We conclude with validation of the model and discussion of its implications and extensibility to other electrolytes.

## 2 Methods and Results

Microscopic interactions between ions and solvents in battery electrolytes govern macroscopic transport properties, and atomistic simulations are ideal for capturing these interactions. Modern computational resources allow atomistic MD simulations of electrolytes to comfortably exceed the relevant time scales for evaluating transport properties.^9,10,12^ In this work, we use atomistic simulations to explore a typical lithium-ion battery electrolyte: 1.5 M lithium hexafluorophosphate (LiPF_6_) dissolved in an equimolar mixture of dimethyl carbonate (DMC) and ethylene carbonate (EC).

We use Gromacs 2020.5 for all molecular dynamics simulations, with OPLS-AA-based force field parameters optimized in our previous work.^10^ We use a background dielectric constant of ϵ = 2 to approximately account for strong local polarization as in our previous work.^10,11^ All bonds of hydrogen atoms are constrained using LINCS,^17^ which enables a time step of 2 fs. We use a Verlet cutoff^18^ of 1.2 nm and compute the long-range electrostatics with the particle-mesh Ewald method.^19^ We use a velocity rescaling thermostat (v-rescale) with a time constant of 1 ps and temperature of 300 K^20^ and a Berendsen barostat with a time constant of 10 ps and pressure of 1 bar.^21^

### 2.1 Mobility Matrix

The Onsager mobility matrix relates the forces acting on the electrolyte species to the molecular fluxes. In our previous work, we measured the drift velocity *v*_*i*_ of species *i* in response to a force per volume *F*_*j*_ on species *j* and defined the mobility matrix *M*_*ij*_ as:^10^

1 $$v_{i}=M_{ij}F_{j}$$

In this work, it is more convenient to consider the force per molecule *f*_*j*_, so we define a mobility matrix *L*_*ij*_ that relates *f*_*j*_ to the molecular flux *J*_*i*_ = *c*_*i*_ *v*_*I*_ (where *c*_*i*_ is concentration of *i*th species) as:

2 $$J_{i}=L_{ij}f_{j}$$

Because *f*_*j*_ = *F*_*j*_/*c*_*j*_ is the force per molecule on the *j*th species, *L*_*ij*_ can be related to *M*_*ij*_ as *L*_*ij*_ = *c*_*i*_ *M*_*ij*_ *c*_*j*_, which likewise satisfies Onsager symmetry *L*_*ij*_ = *L*_*ji*_.

We use nonequilibrium simulations to calculate the mobility matrix of the electrolyte: 1.5 M (equivalently 0.9 nm^–3^) LiPF_6_ dissolved in an equimolar mixture of DMC and EC. We pull one species with a constant force and observe the resulting drift of all the species, as described in our previous work.^10^ The measured mobility matrix *L*_*ij*_ is:

3 $$L_{ij}=\left[\begin{array}{llll} 6.59 & 3.08 & -5.97 & 0.20\\ 3.09 & 6.47 & -7.64 & -3.51\\ -5.69 & -7.25 & 41.08 & -28.49\\ 0.22 & -3.48 & -28.98 & 34.90 \end{array}\right]\times 10^{-2}\;mol\;nm^{-1}\;ns^{-1}\;kJ^{-1}$$

Here, the row and column indices correspond to Li^+^, PF_6_^–^, DMC, and EC, respectively. The unfamiliar units for *L*_*ij*_ follow from the units for $J_{i}=c_{i}v_{i}∼\frac{1}{nm^{3}}\frac{nm}{ns}$ and $f∼\frac{kJ/mol}{nm}$ .

The measured mobility matrix *L*_*ij*_ is symmetric within computational error, as expected from Onsager reciprocity. It also annihilates the mass vector of the electrolyte, that is, *L*_*ij*_·*m*_*i*_ = 0, where *m*_*i*_ is the molar mass of species *i*. This enforces the requirement of no response to a uniform acceleration of the system, which we expect to hold for measurements in the COM frame.

### 2.2 Thermodynamic Susceptibility

Thermodynamic driving forces *f*_*i*_^thermo^ result from chemical potential gradients:

4 $$f_{i}^{thermo}=-\nabla \mu_{i}$$

Here, μ_*i*_ is the chemical potential, which in general depends on concentration of all species. In concentrated multicomponent mixtures such as lithium-ion battery electrolytes, concentration gradients of one species can result in thermodynamic driving forces on other species. For example, the preferential solvation of lithium ions in EC over DMC^10^ causes lithium ions to partition in the EC-rich regions of the simulation box. In general, expanding eq 4 to first order in δ*c*_*j*_ gives off-diagonal terms.

The contribution of concentration variations δ*c*_*j*_ of species *j* to the thermodynamic force *f*_*i*_^thermo^ on species *i* is given by the thermodynamic susceptibility matrix **Λ**:

5 $$f_{i}^{thermo}=-\Lambda_{ij}^{-1}\nabla c_{j}$$

For an external potential ϕ_*j*_ acting on species *j* in the electrolyte, the concentrations of all the species are affected:

6 $$\nabla c_{i}=\Lambda_{ij}\nabla \phi_{j}$$

which is identical to eq 5. We cannot control concentration perturbation δ*c* in simulations; therefore, to determine the susceptibility matrix Λ_*ij*_, we measure the concentration perturbation δ*c*_*i*_ that result from applied potentials δϕ_*j*_(*z*). The thermodynamic forces balance the applied potential as:

7 $$f_{i}^{thermo}=-\nabla \phi_{i}\left(z\right)$$

To measure the susceptibility matrix, we apply weak cosine potentials of the form $\delta \phi_{j}\left(z\right)=\phi_{j}^{0}\;\cos\left(2\pi z/l_{z}\right)$ because cosine potentials are consistent with periodic boundary conditions, and weak cosines produce cosinusoidal concentration variations, which are conveniently fitted to eliminate noise in other Fourier modes.

To perform these simulations, we randomly place 1000 DMC, 1000 EC, and 250 LiPF_6_ molecules in a 6 × 6 × 10 nm^3^ box and equilibrate using semi-isotropic pressure coupling at 1 bar to maintain the box length in *z*-direction. The equilibrated box dimensions are 5.28 × 5.28 × 10 nm^3^, with an average salt concentration of about 0.9 nm^–3^ ≈ 1.5 M.

To perturb the concentration profiles, we apply the potential δϕ_*i*_(*z*) to each molecule of species *i* individually. We use a customized version of Gromacs 2020.5, to which a cosine potential has been added.

To evaluate the full susceptibility matrix for the four-component electrolyte, we perform four simulations, with cosine potentials applied to each of the four species. We choose a potential of strength $\phi_{i}^{0}=0.5\;kJ/mol$ , which is low enough to ensure a linear response. This method is analogous to our measurement of the mobility matrix: instead of applying constant forces and measuring the drift response, we apply a cosine potential and measure the concentration response.

Figure 1 shows the equilibrium concentration profiles for each of the four simulations. Cross correlations are apparent: LiPF_6_ tends to follow EC; and as the electrolyte is nearly incompressible, DMC is anticorrelated to LiPF_6_. This solvent response is caused by the favorable solvation of Li^+^ in EC compared with DMC. The cosine fits of concentration profiles are shown as dotted curves; their amplitudes are recorded as the response in each simulation.

**Figure 1:**
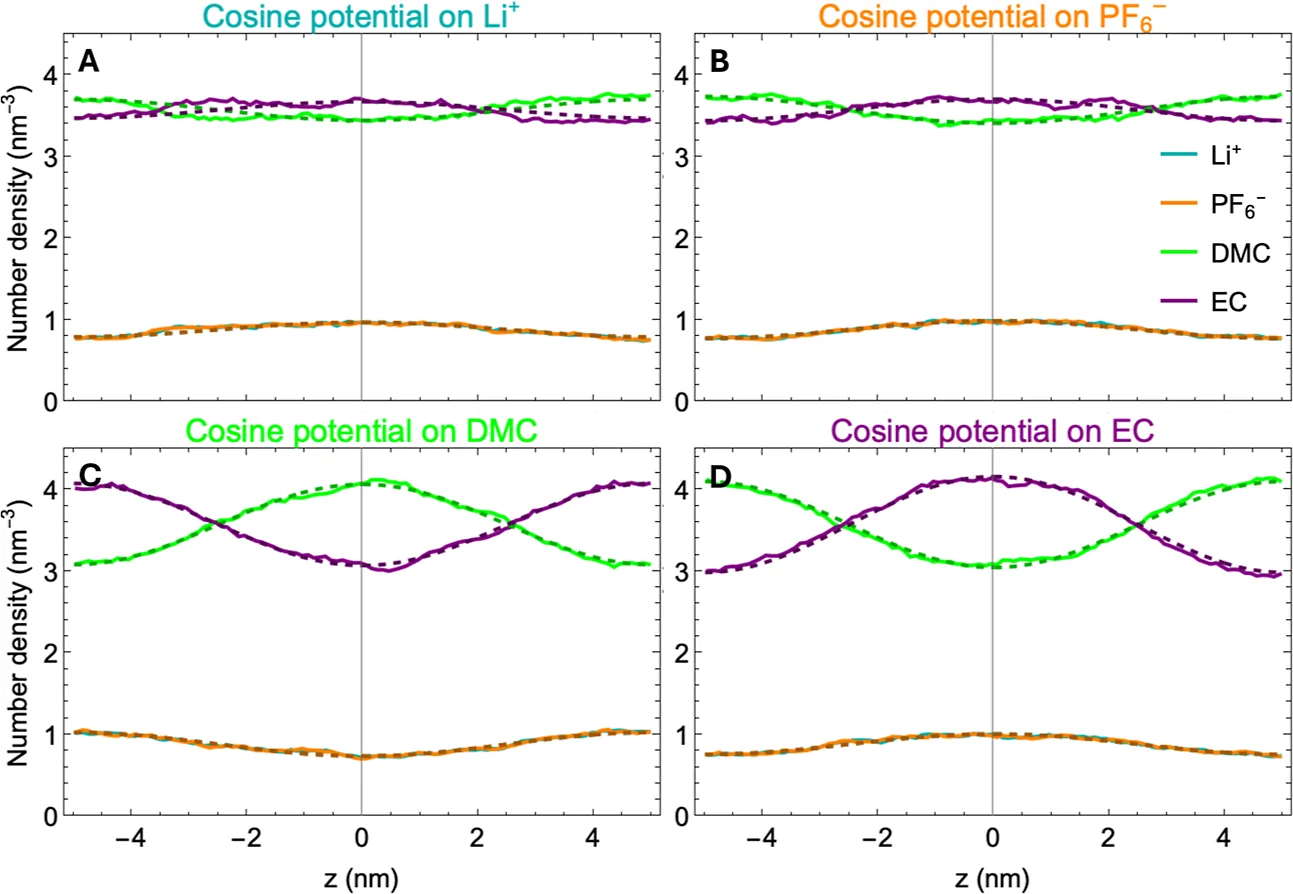
Equilibrium concentration profiles of concentration force calibration simulations with cosine potentials on (A) Li^+^ ions, (B) PF_6_^–^ ions, (C) DMC, and (D) EC. The dotted curves are cosine fits to the concentration profiles. Li^+^ and PF_6_^–^ curves are overlapping.

The Li^+^ and PF_6_^–^ concentrations are observed to be equal everywhere; that is, the electrolyte is neutral everywhere. This suggests the concentration gradient forces are negligible compared to electrostatic forces, which enforce electroneutrality even when we apply potentials only to one of the charged species. Likewise, liquid electrolytes have high bulk moduli and are nearly incompressible: even when we apply potentials to only one species, the volumetric strain remains nearly constant.

Electroneutrality and incompressibility imply that the susceptibility matrix must give no response to cosine potentials corresponding to electric field and uniform compression. To enforce this on the measured susceptibility matrix, we project it to the subspace orthogonal to potential vectors corresponding to an electric field separating ions **a**_**F**_ = [1, −1, 0, 0] and to volumetric compression **a**_**P**_^′^ = [*v*_Li^+^_, *v*_PF_6_^–^_, *v*_DMC_, *v*_EC_] (where *v*_*i*_ is the molecular volume of species *i* in the simulation), such that

8 $$\Lambda^{-1}\cdot a_{F}=\Lambda^{-1}\cdot a_{P}^{\prime }=0$$

The details of calculation of susceptibility matrix, including its manipulation to enforce the assumptions of electroneutrality and incompressibility in eq 8 are described in the Appendix.

The final susceptibility matrix obtained from these simulations is:

9 $$\Lambda^{-1}=\left[\begin{array}{llll} 1.600 & 1.600 & -0.143 & -0.887\\ 1.600 & 1.600 & -0.143 & -0.887\\ -0.143 & -0.143 & 0.196 & -0.152\\ -0.887 & -0.887 & -0.152 & 0.785 \end{array}\right]kJ\;mol^{-1}\;nm^{3}$$

in which the rows and columns correspond to Li^+^, PF_6_^–^, DMC, and EC, respectively. **Λ** ^–1^ has been symmetrized to respect Onsager reciprocity. The rows and columns corresponding to Li^+^ and PF_6_^–^ are identical because of electroneutrality: the salt behaves as one component for calculating concentration gradient forces.

To highlight the importance of nonideal thermodynamic interactions, we can write an analogous susceptibility matrix Λ_*ij*_^′–1^ = δ_*ij*_ *k*_B_ *T*/*c*_*i*_ (δ_*ij*_ is Kronecker delta), which treats the electrolyte as an ideal solution, and we further enforce the assumptions of incompressibility and electroneutrality as described in the Appendix:

10 $$\Lambda^{\prime -1}=\left[\begin{array}{llll} 1.023 & 1.023 & -0.331 & -0.264\\ 1.023 & 1.023 & -0.331 & -0.264\\ -0.331 & -0.331 & 0.373 & -0.251\\ -0.264 & -0.264 & -0.251 & 0.493 \end{array}\right]kJmol^{-1}nm^{3}$$

**Λ′** ^–1^ is significantly different from **Λ** ^–1^. For example, a comparison of the off-diagonal couplings between ions and solvents shows that the marked preference of ions for EC versus DMC in eq 9 is absent in the ideal-solution approximation eq 10. The effects of these differences on the resulting species fluxes are in the Discussion section.

### 2.3 Compressibility

In our battery discharge simulations, a linear compression is observed along the length of the battery (Figure 2A). This compression is caused by repeated transfer of cations to the negative electrode; the resulting gradient of the salt concentration is shown in Figure 4. To account for the pressure difference arising from this compression, we measured the bulk modulus of the electrolyte.

**Figure 2:**
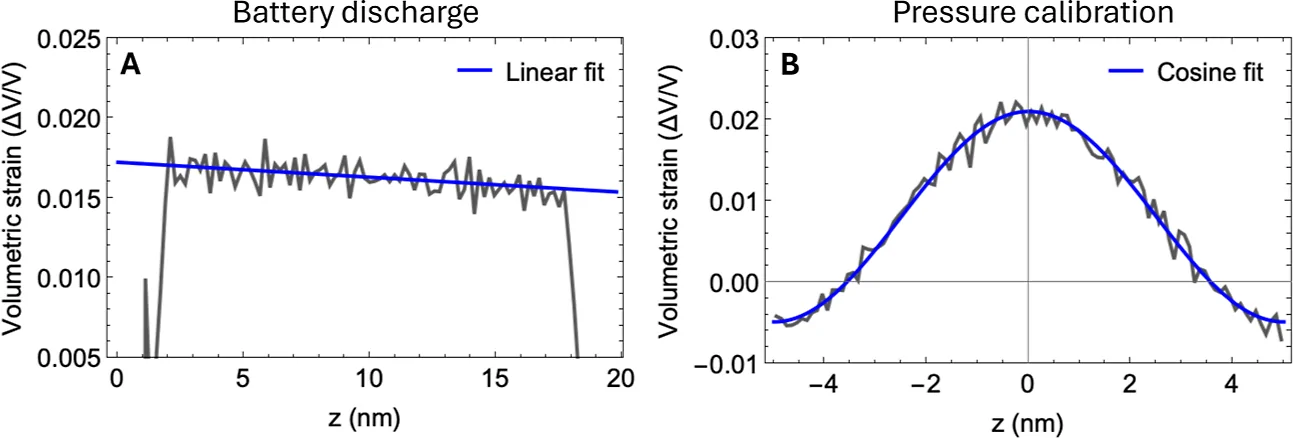
Volumetric strain as a function of the *z*-coordinate of the simulation box in (A) battery discharge simulation and (B) pressure calibration simulation.

Cosine potentials proportional to the molecular volume of the species applied to all molecules, corresponding to a cosinusoidally varying pressure along the length of the simulation box, produces a cosine deviation in volumetric strain as shown in Figure 2B. The molecular volumes of all species are measured in separate simulations at 300 K and 1 bar. The volume of DMC is calculated as a pure fluid, the volume of EC is deduced from volume of equimolar mixture of the two solvents, the volume of PF_6_^–^ is calculated from simulation of 1.5 M charged solution of PF_6_^–^ in equimolar DMC and EC, and the volume of Li^+^ is calculated as the difference between the volume of simulations of the electrolyte and the volume of other electrolyte species (see Table 1). The volume of Li^+^ ions turned out to be negative because adding them to the electrolyte mixture reduces the total volume as a result of strong Coulomb interactions with PF_6_^–^ and the solvents.

**Table 1:** Molecular Volume of Electrolyte Species at 300 K and 1 bar and Bulk Concentrations in the Battery Simulation

species	molecular volume (nm^3^)	concentration (nm^–3^)
Li^+^	–0.0170	0.91
PF_6_^–^	0.0942	0.91
DMC	0.1461	3.63
EC	0.1156	3.60

The bulk modulus is calculated from

11 $$\phi =\Delta P=-\frac{\Delta V}{V}B$$

where Δ*V*/*V* is the volumetric strain and Δ*P* is the compressive stress applied by cosine potentials ϕ. The measured bulk modulus is

12 $$B=1226\;kJ\;mol^{-1}\;nm^{-1}\approx 2\;GPa$$

### 2.4 Battery Discharge

When a battery discharges, cations drift and transport charge between the electrodes across the electrolyte. Our simulation replicates this migration by removing cations from near the positive electrode (right-hand wall) and inserting them near the negative electrode (left-hand wall), thereby maintaining a steady flux of cations across the system.

To prepare the simulation, we randomly insert 1100 DMC, 1000 EC, and 250 LiPF_6_ molecules in a simulation box of dimensions 4 × 4 × 20 nm^3^. Then, we elongate the box to 25 nm in the *z*-direction to avoid overlap of the atoms with the electrodes, which we represent as walls at *z* = 0 nm and *z* = 25 nm. The wall potential is the repulsive part of a Lennard-Jones (LJ) potential function integrated over the wall surface (i.e., 10–4 wall potential), with a particle density of 300 nm^–2^. The LJ parameters for the wall emulate alkane carbon atoms: σ_w_ = −0.35 nm and ϵ_w_ = 0.276144 kJ mol^–1^ (negative σ_w_ sets the attractive part of LJ potential to zero).

We equilibrate this configuration with a 2 ns semi-isotropic simulation at 100 bar and 300 K, allowing the simulation box to contract only along the *z*-axis to maintain a constant wall area. The equilibrated configuration measures 4 × 4 × 19.8 nm^3^, with a bulk electrolyte density around 1285 kg/m^3^. Figure 3 displays the resulting configuration of the equilibrated electrolyte sandwiched between two repulsive walls.

**Figure 3:**
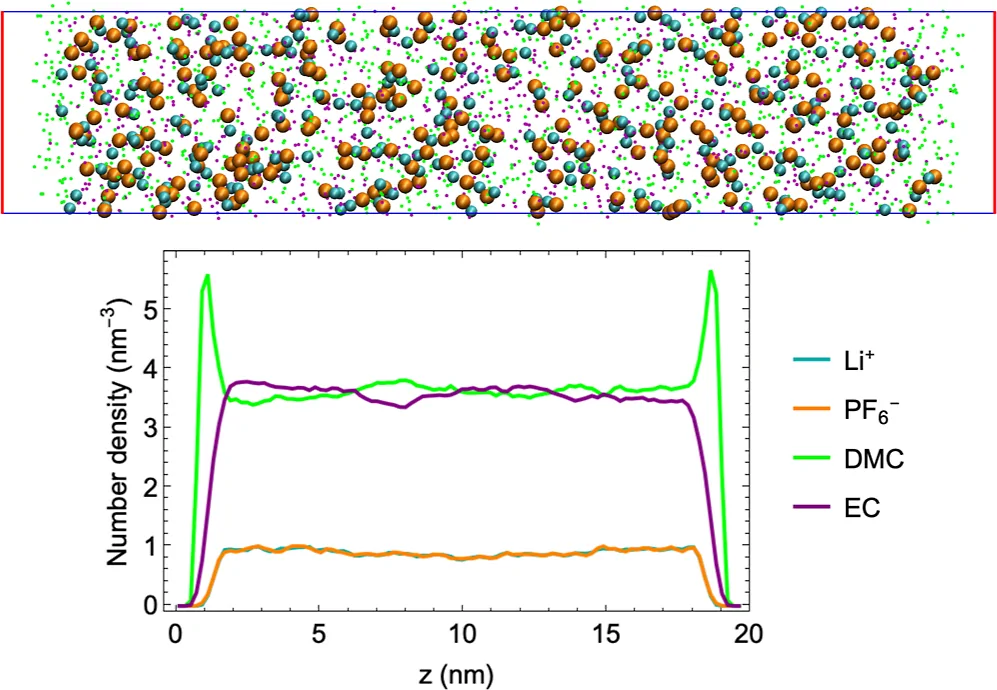
Top: equilibrated configuration of battery simulation. Red boundaries are repulsive walls normal to *z* and blue lines are periodic boundaries in the transverse directions. Bottom: concentration profile of the equilibrated configuration.

Repulsion between the walls and the electrolyte gives rise to a 0.5 nm vacuum gap (Figure 3). The lower polarity of DMC relative to EC results in higher DMC concentration near the walls; the ions prefer to be solvated and thus avoid the walls. This uneven distribution of solvent near the walls results in equal bulk concentrations of the solvents between *z* = 2 nm and *z* = 18 nm.

In a discharging battery, the positive electrode absorbs lithium ions from the electrolyte, and the negative electrode releases an equal number of lithium ions into the electrolyte. To simulate this absorption and release, we periodically remove some of the lithium ions near the positive (right-hand) electrode and insert them next to the negative (left-hand) electrode. We define the region near the positive electrode (the last 15% of the length of the simulation box) as the “warning track”—lithium ions in the warning track have some probability of disappearing and instantaneously appearing in the vacuum region next to the negative electrode, 0.35 nm from the wall.

To execute this ion transfer step, we stop the simulation, alter the *z*-coordinate of lithium ions to be transferred, and continue the simulation with the updated configuration. Frequent ion transfer steps would reduce the computational efficiency, as each step requires stopping and restarting the simulation; too low of a frequency would result in a pulsating ion current. We found that one transfer step every 2 ns works well, with a total simulation time of 2000 ns.

The probability *p* of transferring a lithium ion in the warning track controls the steady-state ionic current density; higher *p* results in higher ionic flux. The value of *p* should be chosen to be low enough to produce a linear response in ionic current. As shown in Figure 4A, *p* = 0.05 is at the edge of the linear response regime but gives easily measured deviations from equilibrium, so we choose this value for our discharge simulation.

**Figure 4:**
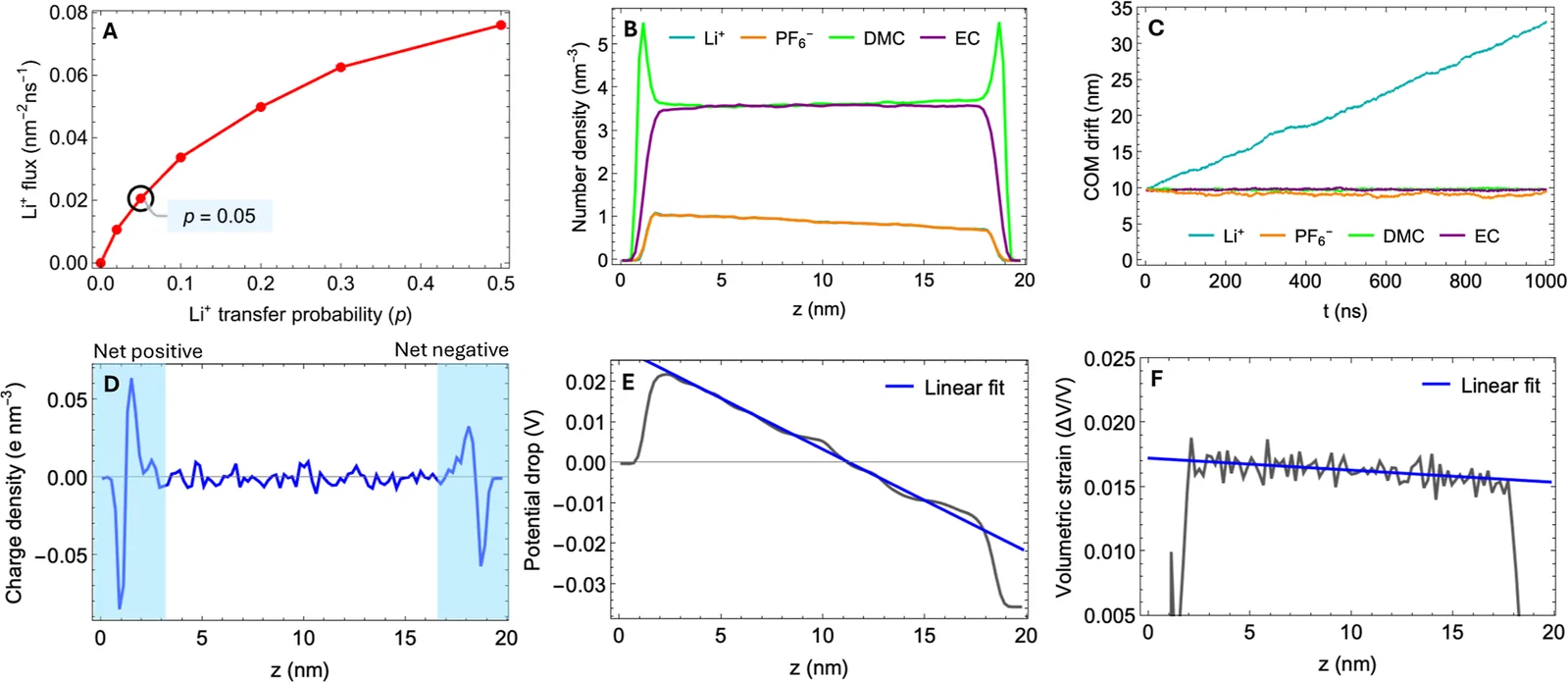
battery discharging simulation at the steady state: (A) response of Li^+^ flux with transfer probability *p*; (B) concentration profiles at *p* = 0.05; (C) Li^+^ drifting at constant speed, (D) charge density of ions (Li^+^ and PF_6_^–^); (E) internal electric potential drop across the electrodes; and (F) volumetric strain arising from repeated Li^+^ insertion near *z* = 0.

On repeated transfer of lithium ions to the negative electrode, a constant concentration gradient of LiPF_6_ develops across the battery at the steady state (see Figure 4B). Away from the electrodes, the battery electrolyte is electrically neutral, with equal concentrations of Li^+^ and PF_6_^–^.

The lithium ions drift with a constant velocity across the electrolyte from the negative electrode to the positive electrode (see Figure 4C). The other species (PF_6_^–^, DMC, and EC) cannot be absorbed or released by the electrodes; therefore, they do not drift. Predicting these drift velocities from the forces acting on the species is a stringent test of our microscopic constituent relations.

The repeated transfer of lithium ions gives rise to a net positive charge near the negative electrode and a corresponding net negative charge near the positive electrode, while the bulk of the battery remains neutral (see Figure 4D). These charges accumulated near the electrodes and give rise to a steady electric field in the bulk electrolyte inside the battery, pushing Li^+^ toward the positive electrode. Figure 4E shows the corresponding linear potential drop across the bulk electrolyte.

The electric field in the bulk is screened by dielectric constant of the electrolyte ϵ_r_, which we calculate by polarizing the electrolyte under an electric field of strength *E* = 0.0625 V/nm and a uniform background dielectric constant ϵ_r_^′^ = 2 for 200 ns:^22^

13 $$\epsilon_{r}=1+\frac{\left\langle M_{total}\right\rangle }{\epsilon_{r}^{\prime }\epsilon_{0}VE}=8.5$$

Here, ⟨*M*_total_⟩ is the total polarization of the electrolyte of volume *V*. The ions Li^+^ and PF_6_^–^ do not directly contribute to ⟨*M*_total_⟩ because of their zero permanent dipole moment but hinder the fluctuation of dipoles of the solvents DMC and EC.

Experimentally, the dielectric constant of pure DMC is 3.2 at 298 K and pure EC is 90 at 313 K (EC is solid at room temperature), while an equimolar mixture of DMC and EC has a dielectric constant of 31.4 at 313 K.^23^ On adding high concentration of LiPF_6_, we expect the dielectric constant to reduce significantly because of solvation effects and Coulomb interactions.^24,25^ Therefore, the effective dielectric constant of our electrolyte ϵ_r_^′^ϵ_r_ = 2 × 8.5 = 17 aligns with the experiments.

The electric field *E* in discharge simulation resulting from net charge near the electrodes is given by Gauss’s law:

14 $$E\left(z\right)=\int_{0}^{z}\frac{\rho \left(z\right)}{\epsilon_{r}^{\prime }\epsilon_{r}\epsilon_{0}}dz$$

Here, ρ­(*z*) is the ion charge density and ϵ_0_ is the permittivity of free space. The resulting electric force on the *i*th species of the electrolyte is:

15 $$f_{i}^{elec}=q_{i}E$$

where *q*_*i*_ is the charge on *i*th species and *E* is the average electric field in the bulk of the electrolyte.

The repeated Li^+^ transfers also give rise to volumetric compression, which creates a pressure gradient. Figure 4F shows the linearly varying compressive strain calculated at the steady state. The compressive force caused by this pressure gradient is given by:

16 $$f_{i}^{comp}=\frac{dP}{dz}v_{i}=-B\frac{d\left(\Delta V/V\right)}{dz}v_{i}$$

where *f*_*i*_^comp^ is the compressive force acting on a molecule of *i*th species, *P* is the pressure given by eq 11, and *v*_*i*_ is the molecular volume of *i*th species.

At the steady state, a constant Li^+^ flux from the negative to the positive electrodes is maintained. Salt concentration, pressure, and electric potential gradients develop between the electrodes, which generate forces on all of the species. Our challenge is to predict the flux of each species from the microscopic constitutive relations and transport coefficients we have separately measured and compare those predictions to the observed fluxes.

## 3 Discussion

In the previous section, we described the origin of the forces acting on the four species of the electrolyte at the steady state in battery discharge simulations and described the computational methods to evaluate them. Figure 5A decomposes the force on each component into contributions from electrostatic forces (eq 15), chemical potential gradients (eq 5), and pressure gradients (eq 16).

17 $$f_{i}=f_{i}^{elec}+f_{i}^{thermo}+f_{i}^{comp}$$

18 $$=q_{i}E-\Lambda_{ij}^{-1}\nabla c_{j}-\frac{dP}{dz}v_{i}$$

Then, applying the Onsager mobility *L*_*ij*_ (eq 2) to the total force per molecule *f*_*j*_ we predict the molecular fluxes, which are plotted with black filled markers in Figure 5B.

**Figure 5:**
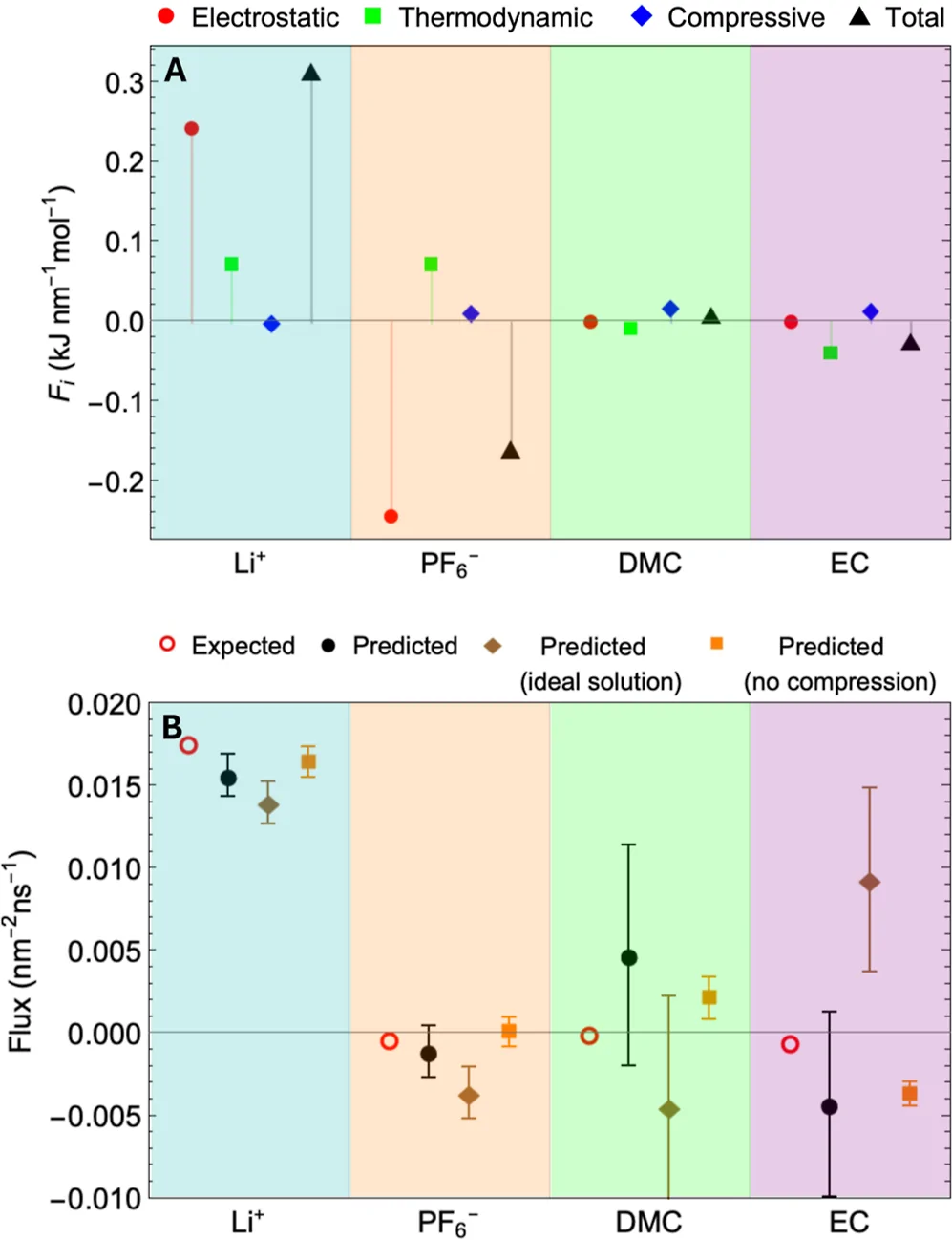
battery discharging simulation at the steady state: (A) forces acting on the electrolyte components and (B) fluxes of electrolyte species from the battery discharge simulations (red open markers) and predictions made using forces and mobility matrix (filled markers). All quantities are measured in center of mass frame.

Electrostatic forces are the main contributors to the total forces on the ions and primarily drive the Li^+^ flux but do not act on neutral solvent molecules. Thermodynamic forces arising from concentration gradients push the ions down the concentration gradient; solvents experience thermodynamic forces only because of their interactions with the ions as their own concentration gradients are essentially zero. Compressive forces arising from the pressure gradient are smallest among the three because of the small volumetric strain gradient in the discharge simulation.

The total force on each species is shown in Figure 5A in black. These forces must be balanced by drag between the drifting Li^+^ and the remaining confined species, resulting in zero flux for everything but the Li^+^ ions. Li^+^ ions have the biggest force pushing them toward the positive electrode; PF_6_^–^ ions experience a significant force in the opposite direction, but the thermodynamic force and the drag from Li^+^ ions keep them from moving. In contrast, the two solvents experience very small forces, primarily arising from off-diagonal thermodynamic interactions and compressive forces.

The large error bars in the predicted fluxes of the two solvents arise from error in measuring the volumetric strain gradient (Figure 2A), as compressive forces significantly affect the solvents. Ignoring the compressive forces reduces the error in fluxes (see Figure 5B), improving the overall results. Therefore, like real batteries, compressive forces can be ignored in these simulations.

In Figure 5B, the predicted fluxes of DMC and EC deviate in opposite directions relative to the expected fluxes because of statistical errors. These deviations may be addressed by combining the two solvents into one species for calculating molecular forces, mobility matrix, and fluxes, resulting in better agreement with expected flux of solvent. Despite these considerations about ignoring the compressive forces and combining the solvents into one species, the molecular fluxes calculated from the mobility matrix agree with those observed in battery discharge simulations, validating our approach.

The off-diagonal terms in the mobility matrix quantify the correlated motion of species and are instrumental in predicting the fluxes in concentrated electrolytes.^9,10,12^ Ignoring the cross-correlations between motion of species is equivalent to relating the flux to the molecular forces at infinite dilution and results in wrong predictions because of miscalculation of drag between species.

Likewise, the nonideal thermodynamic behavior of the electrolyte is quantified by the susceptibility matrix **Λ** ^–1^. The importance of nonideal thermodynamic interactions is apparent in differences between **Λ** ^–1^ and **Λ′** ^–1^ (eqs 9 and 10). Figure 5B also shows flux of each species while ignoring the nonideal thermodynamic interactions calculated using **Λ′** ^**–1**^ in brown diamonds. We find the fluxes of Li^+^ and PF_6_^–^ are lower than expected, while the fluxes of the solvent deviate significantly compared to nonideal predictions and expected values. 10–20% deviations in predicted Li^+^ flux will translate directly to an error of similar magnitude in the predicted discharge rates and internal potential drop of the battery. These significant deviations prove the importance of nonideal thermodynamic interactions in battery electrolytes and demonstrate they must be accounted for in models predicting battery behavior.

The internal potential drop in lithium-ion batteries depends mainly on internal resistance and discharge rate, which typically is 50–100 mV for moderate loads.^26,27^ This range is comparable to our discharge simulations, for which the internal potential drop is about 35 mV (see Figure 4E). In contrast, the typical separation between electrodes of real lithium-ion batteries is 10–100 μm or about 3 orders of magnitude larger than our discharge simulations.^27^ This smaller distance results in steeper potential, concentration and pressure gradients, and consequently larger total forces in our simulations compared to real batteries. Although the forces are smaller in real batteries, the relative contribution from each kind of force will be similar. Thus, our microscopic simulations, which are at the edge of linear response, are relevant to the behavior of real battery electrolytes.

## 4 Conclusion

We developed a model of discharging lithium-ion batteries at atomistic detail. We model an electric cell as an electrolyte sandwiched between parallel electrodes: the positive electrode takes up the cations and the negative electrode expels cations at the same rate, replicating battery discharge.

This simple transfer of cations, in an otherwise equilibrium molecular dynamics simulation, brings about several physically relevant effects at the steady state that are crucial to fundamentally understanding battery operation. At the steady state, the electric current-carrying cations attain a constant flux across the electrolyte from the negative to the positive electrode. This flux is primarily driven by three forces on the electrolyte: the electrostatic force, forces due to concentration gradients, and compressive forces, all arising from the transfer of cations at the electrodes.

We evaluate these forces using separate calibrating simulations and relate them to the observed fluxes in the battery using mobility matrices. This work unifies four different sets of simulations: (1) the battery discharging simulations for electrostatic forces, (2) the simulations for calibrating the concentration gradient forces, (3) the compression calibration simulation, and (4) the simulations for measuring the mobility matrix to demonstrate battery operation at atomistic detail for the first time.

We decompose the force on each species into electric forces arising from charge accumulation at the electrodes, thermodynamic forces arising from chemical potential gradients, and compressive forces arising from the pressure difference. This work also highlights the importance of accounting for the nonideal thermodynamic interactions between the species and their correlated motion in making crucial predictions related to battery discharge.

This work focuses on the forces developed in the bulk electrolyte during discharging and does not take into account the impedance at the electrode–electrolyte interface. In real batteries, interfacial impedance will introduce an additional voltage drop in proportion to the ionic flux. The interfacial capacitance could not be reproduced in these simulations.

We have demonstrated this model on typical organic liquid-based lithium-ion batteries, but these methods can be extended to any materials that can be modeled using molecular dynamics simulations, including polymer and aqueous electrolytes. Further, this model differentiates the origin of forces acting on each component of the electrolyte, aiding a better physical and quantitative understanding of battery operation.

This model may be extended to incorporate battery charging by applying a potential difference across the electrodes and observing the resulting drift of the cations from the positive electrode to the negative electrode.

## Appendix

### Calculating Thermodynamic Susceptibility Matrix

Let the components of the electrolyte, i.e., Li^+^, PF_6_^–^, DMC, and EC be numbered 1 through 4, respectively. The susceptibility matrix for this electrolyte, **Λ** has dimensions 4 × 4, with the rows and columns corresponding to each electrolyte species.

In the susceptibility matrix simulations described in the Methods and Results section, the amplitudes of the cosine potentials are the inputs producing the responses, which are the amplitudes of the equilibrium cosine fits of concentration profiles. The inputs are *a*_*ij*_ = δ_*ij*_, and the recorded the responses are *r*_*ij*_, where δ_*ij*_ is the Kronecker delta, *i* is the index of the species with cosine potential, and *j* is the species index. For example, for the simulation with cosine potential on the third species DMC, **a**_**i**_ = [0, 0, 1, 0] and **r**_**i**_ = [*r*_31_, *r*_32_, *r*_33_, *r*_34_].

As justified in the Methods and Results section, we want to project the thermodynamic response of the electrolyte on a subspace orthogonal to the vectors corresponding to electric field and pressure. For that, we define four principal vectors: **a**_**F**_ = [1, −1, 0, 0] corresponding to electric field, **a**_**P**_^′^ = [*v*_1_, *v*_2_, *v*_3_, *v*_4_] corresponding to pressure (*v*_*i*_ is molecular volume of species *i*), **a**_**E**_ = [0, 0, *v*_4_, −*v*_3_] the solvent exchange vector, and $a_{R}=\left[1,1,\frac{-v_{3}\left(v_{1}+v_{2}\right)}{\left(v_{3}^{2}+v_{4}^{2}\right)},\frac{-v_{4}\left(v_{1}+v_{2}\right)}{\left(v_{3}^{2}+v_{4}^{2}\right)}\right]$ , pushing the salt to the center. **a**_**E**_ and **a**_**R**_ are chosen such that they are orthogonal to **a**_**F**_ and **a**_**P**_^′^.

All pairs of these principal vectors are mutually orthogonal except for **a**_**F**_ and **a**_**P**_^′^. To fix that, we subtract the component of **a**_**P**_^′^ along **a**_**F**_:

19
$$a_{P}=a_{P}^{\prime }-a_{F}\frac{a_{P}^{\prime }\cdot a_{F}}{a_{F}\cdot a_{F}}$$

Henceforth, we will use the unit principal vectors, denoted as ${â}_{i}$ .

We construct a matrix **N** as the sum of the dyadic products of responses **r**_**i**_ and input potentials **a**_**i**_:

20
$$N=\sum_{i=1}^{4}r_{i}a_{i}^{T}$$

Then, we project **N** on the subspace orthogonal to ${â}_{F}$ and ${â}_{P}$ , which is, along ${â}_{E}$ and ${â}_{R}$ :

21
$$r_{E}=N\cdot {â}_{E}$$

22
$$r_{R}=N\cdot {â}_{R}$$

Here, **r**_**E**_ and **r**_**R**_ are the responses along ${â}_{E}$ and ${â}_{R}$ . Then, we construct **Q** on the subspace of ${â}_{E}$ and ${â}_{R}$ :

23
$$Q=r_{E}{{â}_{E}}^{T}+r_{R}{{â}_{R}}^{T}$$

**Q** is the symmetric, positive definite matrix and cannot be inverted normally. Therefore, we construct a projection matrix **P** using dyads of ${â}_{F}$ and ${â}_{P}$ :

24
$$P=I-{â}_{F}{{â}_{F}}^{T}-{â}_{P}{{â}_{P}}^{T}$$

We get an invertible matrix **S** using **P**:

25
$$S=P\cdot Q\cdot P+{â}_{F}{{â}_{F}}^{T}+{â}_{P}{{â}_{P}}^{T}$$

We invert **S** and project it back to the original subspace to construct the inverse susceptibility matrix **Λ** ^**–1**^:

26
$$\Lambda^{-1}=P\cdot S^{-1}\cdot P$$

### Center of Mass Frame of Reference for Discharge Simulations

Figure 5B shows the molecular flux of the four electrolyte species in the battery discharge simulations, and the predicted fluxes from our approach using mobility matrices in center of mass frame. We measure the mobility matrix in center of mass (COM) frame of reference, therefore, it enforces a zero flux for the COM. This condition is violated in battery discharge simulations from repeated transfer of lithium ions. To account for this disparity, we assign molecular fluxes to the confined species of the electrolyte, i.e., PF_6_^–^, DMC, and EC, such that the COM remains at rest:

27
$$\sum_{i}m_{i}J_{i,COM}=0$$

where *m*_*i*_ is the molecular mass of species *i* and *J*_*i*,COM_ is the molecular flux with respect to the COM of the electrolyte.

To calculate molecular fluxes in the COM frame of reference, we calculate the velocity of COM Li^+^ as the slope of drift versus time plot in the battery discharge simulations shown in Figure 4C. Drift velocities of the confined species is zero. The molecular flux with respect to COM *J*_*i*,COM_ is given as:

28
$$J_{i,COM}=c_{i}v_{i,COM}=c_{i}\left(v_{i}-\sum_{i}\rho_{i}v_{i}\right)$$

where ρ_*i*_ is the mass fraction of species *i* in the electrolyte, *c*_*i*_ is the average concentration, and *v*_*i*_ is the COM drift velocity and *v*_*i*,COM_ of species *i* with respect to COM of the electrolyte in battery discharge simulations. Because of these frames of the COM frame of reference, molecular fluxes of confined species are slightly negative in Figure 5B.
